# Chicago Veterinary College

**Published:** 1903-05

**Authors:** 


### CHICAGO VETERINARY COLLEGE.

The nineteenth annual commencement exercises of this college were held at the College Auditorium on Friday evening, March 27, 1903. The Auditorium was handsomely decorated for the occasion, and was filled with an appreciative audience of more than three hundred, numerous ladies, friends of the faculty, and graduating class being present.

Professor A. H. Baker presided, and the following members of the faculty were present: Professors Joseph Hughes, M.R.C.V.S.; A. S. Alexander, F.H.A.S., M.D.C.; E. L. Quitman, M.D.C.; J. F. Ryan, D.V.S.; G. A. Lytle, M.D.C.; G. M. Cushing, M.D.; L. A. Merillat, M.D., D.V.S.; E. Merillat, A.B., M.D.V.; James M. Wright, D.V.S.; James Robertson, M.D.C.; John D. Robertson, M.D., and C. A. White, M.D.C. In a short introductory address, Professor Baker commented on the exceptionally good showing made by the class of 1902–03, complimenting them on their regularity in attendance, general conduct, and high average in their final examinations.

Professor Hughes then read the Secretary's report, congratulating the present class on their high attainments, and stating that in point of quality they have never been surpassed by any previous class. He alluded to the fact that this year was a record breaker both with regard to the number of honorary graduates and regular students attending. The classes of 1902–03 comprised 185 regular students and 20 postgraduates. Of the regular students 35 were from other schools, no doubt attracted by the high character of the course given by this college. He pointed out that the Chicago Veterinary College had this year the largest attendance of any three-year veterinary school on this continent, or for that matter in the English-speaking world. On behalf of the trustees, he wished to publicly thank the faculty for their earnest efforts and devotion to duty during the past session. Efforts such as theirs deserve reward. The large and appreciative classes, who so regularly attended and worked so assiduously under their guidance, and who at final examinations have been so successful, amply compensate them. Concluding, he announced the names of those who had passed the final examinations.

R. M. Allen, Marshalltown, Ia.; J. L. Axby, Guilford, Ind.;

F. N. Anderson, Buffalo, N. Y.; L. F. Barber, Tyndall, N. D.; F. R. Bernard, Sheridan, Ill.; H. P. Boettner, Perryville, Mo.; P. H. Browning, Carrollton, Mo.; D. B. Clark, Janesville, Wis.; A. B. Cox, Chicago, Ill.; C. A. Dawdy, Greenville, Ill.; A. L. Deal, Wilmot, Ohio; C. S. Evans, Grand Island, Neb.; E. O. Faires, St. Jacob, Ill.; O. K. Faires, St. Jacob, Ill.; L. A. Forge, Burlington, Wis.; G. J. Graen, Toulon, Ill.; S. Gault, Knoxville, Ill.; F. A. Gibbs, Palatine, Ill.; H. H. Gibbs, Flora, Ind.; H. B. Hallenberger, Hannibal, Mo.; C. A. Hatterscheid, Corwith, Iowa; H. F. Hisgen, Chicago, Ill.; A. B. Hollis, Des Moines, Iowa; J. L. Hoylman, Wilsonville, Neb.; J. B. Jaffray, Chicago, Ill.; A. Kaderabek, Jr., Delafield, Wis.; W. H. Kenwell, Mt. Eaton, Ohio; G. W. Johnson, Chicago, Ill.; H. J. Kohler, Somerville, N. J.; E. L. Lewis, Opelousas, La.; James Lewis, McKinney, Tex.; D. J. McMahan, Noblesville, Ind.; L. B. McMichael, St. Jacob, Ill.; H. J. Mongeau, Mantens, Ill.; N. C. Nelson, Manistee, Mich.; J. H. Newman, Little Falls, Minn.; C. Olson, Athens, Ill.; G. M. Otis, Des Moines, Iowa; J. M. Parks, Covington, Ky.; L. J. Price, Liberty Center, Ohio; F. E. Perkins, Ellsworth, Wis.; L. H. Quitman, Chicago, Ill.; J. W. Robinson, Coal Harbor, N. D.; G. E, Repp, Chambersburg, Pa.; A. H. Schmoyer, Boynton, Pa.; C. J. Schultz, Columbus, Wis.; R. Snyder, Dixon, Iowa; C. J. Spencer, Jasper, N. Y.; J. F. Spiker, Jr., Chariton, Iowa; C. E. Stockton, Braddock, Pa.; J. F. Talbert, Edgerton, Kas.; W. W. Talbot, Omaha, Neb.; J. L. Ward, Bowling Green, Mo.; C. M. Weise, Plattville, Ill.; G. R. Weise, Princeton, Ill.; M. M. White, Shreveport, La.; W. D. Wright, Walla Walla, Wash.; M. J. Woodliffe, Denver, Col.; B. J. Zimprich, Marshall, Wis.

The following comprise the honor list:

J. L. Axby, C. A. Dawdy, C. A. Hatterscheid, A. B. Hollis, J. B. Jaffray, H. J. Kohler, E. L. Lewis, D. J.McMahan, C. Olson, G. M. Otis, J. M. Parks, L. H. Quitman, J. W. Robinson, A. H. Schmoyer, C. G. Schultz, R. Snyder, C. M. Weise, M. M. White, W. D. Wright, M. J. Woodliffe, B. J. Zimprich.

Following the Secretary's report, Professor A. H. Baker conferred the degree of Doctor of Comparative Medicine (M.D.C.) on the members of the class, and Professors Hughes and Quitman distributed the diplomas.

To Professor L. A. Merillat fell the pleasing duty of distributing the prizes, and as each successful recipient came forward to claim his hard-earned honor he was felicitously addressed by the professor.

Dr. C. G. Schultz received the gold medal for the highest general average, and also the gold medal in anatomy. Dr. Rudolph Snyder gold medal in equine pathology; Dr. B. J. Zimprich, gold medal in cattle pathology; Dr. Carl Olson, gold medal in general surgery; Dr. L. H. Quitman, prize in materia medica; Dr. D. J. McMahan, prize in lameness; Dr. J. L. Axby, prizes in bacteriology and physiology; Dr. C. A. Dawdy, prize in meat and milk inspection; Dr. W. J. Robinson, prize in hygiene; Dr. J. B. Jaffray, prize in canine and feline pathology; Dr. C. A. Dawdy, prize in helminthology, and Dr. L. B. Michael, prize in dentistry.

Dr. A. Kaderabek was class prophet, and Dr. J. L. Axby valedictorian.

In conclusion, Professor A. H. Baker delivered the doctorate, giving the class valuable advice and encouragement, commenting upon the fine prospects of the profession, wishing them Godspeed, and impressing upon them the privilege of turning to their alma mater for future advice if necessary, and wished them to consider every individual member of the faculty as their friend. He urged them to conduct themselves in such a way as to command the respect of the people, and thus uphold the dignity of the profession, and win fame for themselves and reflect credit upon the Chicago Veterinary College.

---

*Bulletin No.* 125 *of the U. S. Department of Agriculture*, relative to "Horse Feeding Experiments," just issued, is a valuable compilation of the results of a long line of experimental tests of this important factor in animal industry.

*Bulletin No.* 44 *of the Bureau of Animal Industry*, U. S. Department of Agriculture, is a very complete exposition on the "Infectiveness of Milk of Cows which have Reacted to the Tuberculin Test," by Dr. John R. Mohler, A.M., V.M.D., Chief of the Pathological Division.

---

Many excellent papers are promised for the meeting at Ottawa, Canada. Secretary Repp is carrying on the work of his office in a very vigorous manner, while our Canadian colleagues are preparing for a royal entertainment of those who will join in the pilgrimage to that noted seat of government.

Dr. C. R. Webber, graduate of Chicago Veterinary College, is associated with his brother at Rochester, N. Y. They conduct a veterinary hospital and livery and boarding stable in connection with their practice.

